# Presence of depression and anxiety with distinct patterns of pharmacological treatments before the diagnosis of chronic fatigue syndrome: a population-based study in Taiwan

**DOI:** 10.1186/s12967-023-03886-1

**Published:** 2023-02-08

**Authors:** Chi Chen, Hei-Tung Yip, Kam-Hang Leong, Wei-Cheng Yao, Chung-Lieh Hung, Ching-Huang Su, Chien-Feng Kuo, Shin-Yi Tsai

**Affiliations:** 1 Department of Psychiatry, National Taiwan University Hospital, Taipei, Taiwan; 2 Management Office for Health Data, China Medical University Hospital, Taichung, 404 Taiwan; 3 Department of Medicine, MacKay Medical College, New Taipei City, 252 Taiwan; 4 Department of Laboratory Medicine, MacKay Memorial Hospital, Taipei, 104 Taiwan; 5 Department of Anesthesiology and Pain Medicine, Min-Sheng General Hospital, Taoyuan, Taiwan; 6 Institute of Biomedical Sciences, MacKay Medical College, New Taipei City, Taiwan; 7 Department of Nursing, Nursing and Management, MacKay Junior College of Medicine, New Taipei City, 25245 Taiwan; 8 Division of Infectious Diseases, Department of Internal Medicine, MacKay Memorial Hospital, Taipei, Taiwan; 9 Institute of Long-Term Care, MacKay Medical College, New Taipei City, Taiwan; 10 Department of Health Policy and Management, Johns Hopkins Bloomberg School of Public Health, Johns Hopkins University, 615 N. Wolfe Street, Baltimore, MD 21205 USA

**Keywords:** Chronic fatigue syndrome, Depression, Anxiety, Pharmacological treatment

## Abstract

**Objective:**

An increased prevalence of psychiatric comorbidities (including depression and anxiety disorder) has been observed among patients with chronic fatigue syndrome (CFS). However, few studies have examined the presence of depression and anxiety disorder before the diagnosis of CFS. This study aimed to clarify the preexisting comorbidities and treatments associated with patients with subsequent CFS diagnosis in a population-based cohort in Taiwan.

**Methods:**

An analysis utilizing the National Health Insurance Research Database of Taiwan was conducted. Participants included were 6303 patients with CFS newly diagnosed between 2000 and 2010 and 6303 age-/sex-matched controls.

**Results:**

Compared with the control group, the CFS group had a higher prevalence of depression and anxiety disorder before the diagnosis of CFS. Sampled patients who took specific types of antidepressants, namely, selective serotonin reuptake inhibitors (adjusted odds ratio [aOR] = 1.21, 95% confidence interval [CI] 1.04–1.39), serotonin antagonists and reuptake inhibitors (SARI; aOR = 1.87, 95% CI 1.59–2.19), and tricyclic antidepressants (aOR = 1.46, 95% CI 1.09–1.95), had an increased risk of CFS. CFS risk was also higher among participants taking benzodiazepine, muscle relaxants, and analgesic drugs. A sub-group analysis revealed that SARI use was related to an increased risk of CFS in the depression, anxiety disorder, male, and female groups. In the depression and anxiety disorder groups, analgesic drug use was associated with an increased CFS risk. Nonpharmacological treatment administration differed between men and women.

**Conclusion:**

This population-based retrospective cohort study revealed an increased risk of CFS among populations with preexisting depression and anxiety disorder, especially those taking SARI and analgesic drugs.

**Supplementary Information:**

The online version contains supplementary material available at 10.1186/s12967-023-03886-1.

## Introduction

Patients with chronic fatigue syndrome (CFS) experience prolonged and disabling fatigue that cannot be explained with the existing state of medical knowledge. The prevalence of CFS differs widely depending on the diagnostic criteria, assessment method, and studied population, with its numbers ranging from 0.2% to 6.41% [1, 2]. A systematic review of 46 studies in 2020 estimated a CFS prevalence rate of 0.89% on the basis of the commonly used Centers for Disease Control (CDC)-1994 definition of CFS [3, 4]. The aforementioned review also reported a sex difference, with female individuals having prevalence rates that were 1.5 to 2 times higher than those of male individuals.

In addition to fatigue, several accompanying symptoms were also frequently reported, specifically muscle pain, multiple joint pain, poor sleep, anxiety, and depression [5]. Musculoskeletal pain and insomnia were included in the CDC-1994 diagnostic criteria. Furthermore, mood and anxiety disorders were reported to be more prevalent in individuals with CFS relative to the general population [6]. CFS, which is also known as myalgic encephalomyelitis, had found to be potentially related with immune processes such as inflammation and infection [7]. Recent comparisons between the similarities of CFS and the potential COVID-19 long-term effects, including persistent fatigue, postexertional malaise and pain, had underlined the critical role of the immune response in such conditions [8, 9]. On the other hand, the systemic inflammation may be the mediator of CFS and its psychiatric comorbidities [10, 11]. It is notable that the relationship between CFS and psychiatric comorbidities might be bidirectional as an abnormal immune response has also been demonstrated among the patients with depression or anxiety disorder [12–14]. A study investigated patients with CFS and reported that the prevalence rates of concurrent anxiety and depression were 42.2% and 33.3%, respectively [15]. However, few large-scale epidemiological investigations of psychiatric comorbidities, especially those that focused on Asian populations, have been conducted.

With a focus on CFS, depression, and anxiety, this population-based retrospective cohort study investigated and analyzed the data from the Taiwan National Health Insurance Research Database (NHIRD). The treatments received by participants were also further analyzed by sex, age, and comorbidities.

## Methods

### Data resource

The dataset used in this study were derived from the National Health Insurance Research Database (NHIRD) in Taiwan. The National Health Insurance (NHI) program was launched on March 1, 1995, by Taiwan’s government. NHIRD has contained details concerning the demographic characteristics, dates of admission and discharge, prescriptions, surgical procedures, and diagnostic codes for approximately 99% of the entire population of the 23 million people residing in Taiwan. We used the 2000 Longitudinal Health Insurance Database (LHID) which was established by NHIRD. LHID 2000 was created and released to the public by NHIRD and includes all the original claim data and registration files between 2000 and 2013 for one million individuals randomly sampled from the Registry for beneficiaries of the NHI program in 2000 in Taiwan. The diseases are defined according to the *International Classification of Diseases, Ninth Revision, Clinical Modification* (*ICD-9-CM*).

### Sample participants

**Fig. 1:**
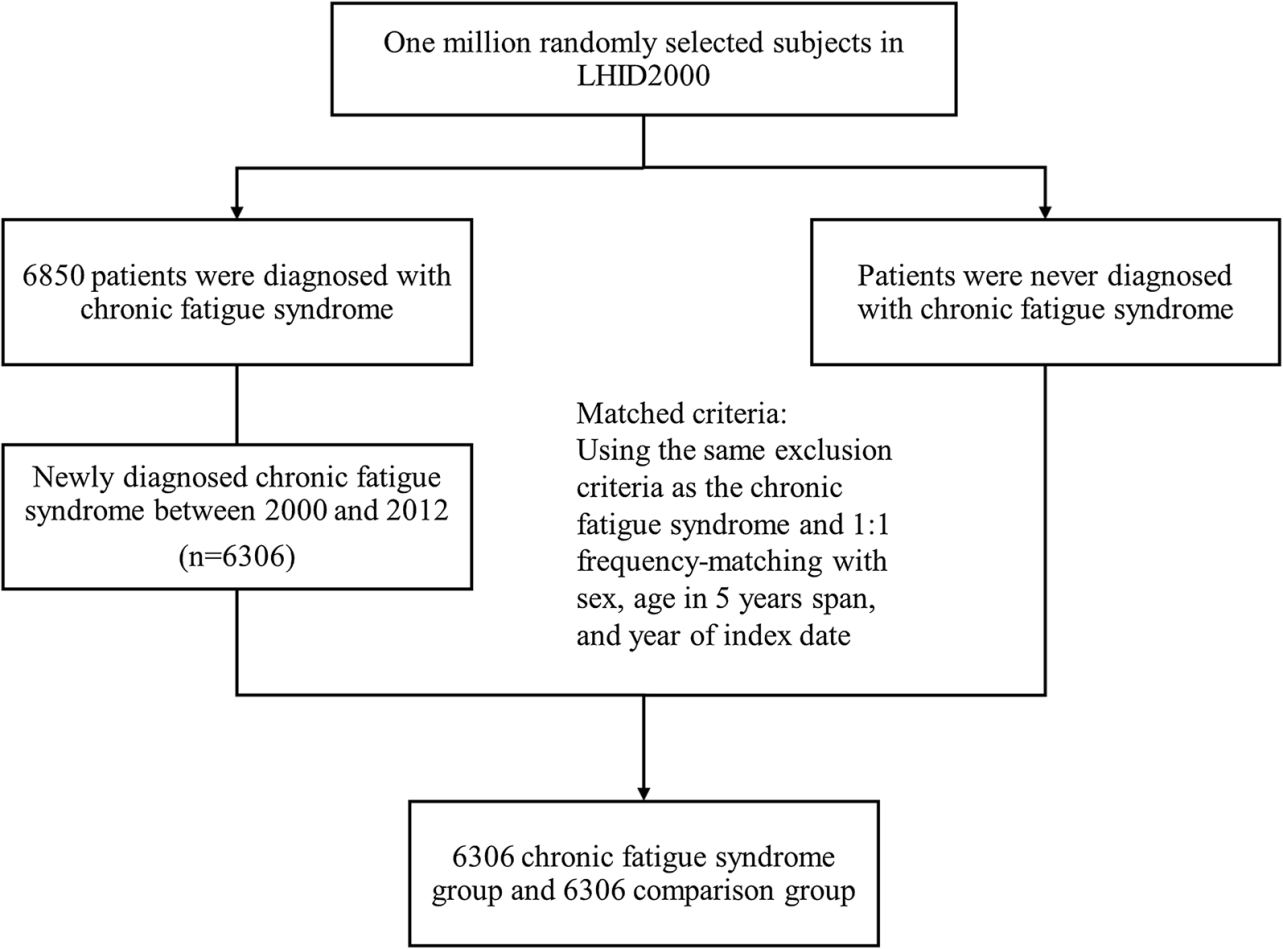
The participants selecting process in the cohort study

Cases of CFS were identified using two outpatient records or one admission record with a diagnosis of *ICD-9-CM* code 780.71. The date of the first diagnosed record of chronic fatigue syndrome was defined as the index date. For each chronic fatigue syndrome case, we used a frequency matching method and randomly selected one control without chronic fatigue syndrome diagnosis. The dataset for the control population of 1 million samples was randomly selected from the LHI dataset, and individuals without a diagnosis of CFS were selected as the control population with the same sex, age, and index date. (Fig. 1.) We excluded the participants aged below 18 years or with missing information on sex. In the ICD-9-CM, the diagnosis of CFS is mainly based on the CDC-1994 diagnostic criteria noted in the ICD-9-CM Coordination and Maintenance Committee Meeting in 2011. The CDC-1994 diagnostic criteria specifically defined the patients receiving appropriate treatment for depression or anxiety, the diagnosis could still be made among patients with premorbid depression or anxiety [3].

### Exposure assessment and comorbidities

For this study, we examined the exposure of pharmaceutical and non-pharmaceutical treatments. We accounted the exposure to pharmaceutical treatments of SSRI drugs (ATC code N06AB10, N06AB06, N06AB03, and N06AB08), SNRI drugs (ATC code N06AX21, and N06AX16), SARI drugs (ATC code N06AX05), norepinephrine and dopamine reuptake inhibitor (NDRI) drug (ATC code N06AX12), noradrenergic and specific serotonergic antidepressants (NaSSA) drug (N06AX11), TCAs drugs (ATC code N06AA09 and N06CA01), BZD drugs (ATC code N03AE01, N05BA06, N05BA12, N05BA01, N05BA17, N05BA22, N05CD04, N05CD05, N05CD03, N05CD09, N05CD01, N05CD08), muscle relaxant (ATC code M03BX08), analgesic drugs which including acetaminophen, NSAIDs, pregabalin, gabapentin (ATC code M02AA, D11AX18, M01A, M01B, N03AX16, and N03AX12) and non-pharmaceutical of support psychotherapy, supportive group psychotherapy, deep psychotherapy, in-depth group psychotherapy, special psychotherapy, special group psychotherapy, behavioral therapy evaluation, behavioral therapy plan, supportive psychosocial consultation for family members/caregivers, stretching exercise, exercise therapy, breathing exercise, induced deep breathing exercise, rehabilitation exercise, multiple physical examinations of sleep, brainwave examination, sleep or wakefulness, and brainwave examination for sleep disorders. Study participants were categorized based on their pharmaceutical and non-pharmaceutical exposure status. Patients exposed to pharmaceutical or non-pharmaceutical were classified as users or non-users. We adjusted for the potentially confounding effects of other comorbidities, including depression (ICD-9-CM code 296.2, 296.3, 926.82, 300.4, 309.0, 309.1, and 311), anxiety disorder (ICD-9-CM code 300.0–300.3, 300.5–300.9, 309.2–309.4, 309.81, and 313.0), insomnia (ICD-9-CM code 307.41, 307.42, 780.50, and 780.52), suicide (ICD-9-CM code E950-E959), Crohn’s disease (ICD-9-CM code 555), ulcerative colitis (ICD-9-CM code 555–556), renal disease (ICD-9-CM code 580–589), diabetes mellitus (ICD-9-CM code 250 and A181), obesity (ICD-9-CM code 278), gout (ICD-9-CM code 274), dyslipidemia (ICD-9-CM code 272), malignancy (ICD-9-CM code 140–208), HIV (ICD-9-CM code 042–044), rheumatoid arthritis (ICD-9-CM code 714), psoriasis (ICD-9-CM code 696.x), ankylosing spondylitis (ICD-9-CM code 720.0), lymphadenopathy (ICD-9-CM code 289.1–289.3, 686, and 785.6), Hashimoto's thyroiditis (ICD-9-CM code 245.2), Sjogren's syndrome (ICD-9-CM code 710.2), irritable bowel syndrome (ICD-9-CM code 564.1), SLE (ICD-9-CM code 710.0), celiac disease (ICD-9-CM code 579.00, and herpes zoster (ICD-9-CM code 053) prior to the index date were evaluated as part of the analysis.

### Statistical analysis

Descriptive statistics of CFS and controls are reported, including demographic characteristics, comorbid diseases, and exposure to potentially confounding treatments. The chi-square test was used to compare categorical variables, whereas the Student’s t-test was used to compare continuous variables between chronic fatigue syndrome cohort and comparison cohort as necessary. We used conditional logistic regression to assess the risk of CFS according to each category of pharmaceutical and non-pharmaceutical. The odds ratio (ORs) and 95% confidence intervals (CIs) for CFS were calculated as an unadjusted incidence rate, and then subsequently adjusted for covariates including age, sex, comorbidities, pharmaceutical and non-pharmaceutical. Bonferroni correction was performed for the correction of multiple comparisons. Analyses were performed using SAS software (version 9.4 for windows; SAS Institute, Cary, NC, USA) for Windows 10. All statistical significance levels were set at a *p* < 0.05.

## Results

This study included 6306 patients with CFS and 6306 patients without, all of whom were identified from the NHIRD between January 1, 2000, and December 31, 2013. The demographic and clinical characteristics of the study participants are presented in Table 1. Among the participants, 52.9 were female, and most were between 25 and 64 years old; the mean age of the participants was 50.6 years. With regard to the prevalence of comorbidities, participants with CFS had higher numbers of psychiatric disorders (depression, anxiety disorder, and insomnia), irritable bowel syndrome, inflammatory bowel diseases (Crohn’s disease and ulcerative colitis), autoimmune disorders (rheumatoid arthritis, and Sjogren’s syndrome), metabolic disorders (type 2 diabetes mellitus, gout, and dyslipidemia), and renal disease (all p < 0.005).

**Table 1:** Demographic characteristics and comorbidities of patients newly diagnosed with or without chronic fatigue syndrome in Taiwan during 2000–2010

Variable	CFS cohort	Non-CFS cohort	P-value
	(n = 6306)	(n = 6306)	
Gender			> 0.99
Female	3339 (52.9)	3339 (52.9)	
Male	2967 (47.1)	2967 (47.1)	
Age at diagnosis of CFS			> 0.99
≤ 34	1350 (21.4)	1350 (21.4)	
35–64	3485 (55.3)	3485 (55.3)	
≥ 65	1471 (23.3)	1471 (23.3)	
Age at diagnosis of CFS(mean, SD)†	50.6 (17.9)	50.6 (18.0)	0.80
Comorbidity			
Depression	807 (12.8)	407 (6.45)	< 0.0001
Anxiety disorder	2038 (32.3)	1033 (16.4)	< 0.0001
Insomnia	2303 (36.5)	1106 (17.5)	< 0.0001
Irritable bowel syndrome	886 (14.1)	423 (6.71)	< 0.0001
Crohn's disease	255 (4.04)	121 (1.92)	< 0.0001
Ulcerative colitis	279 (4.42)	138 (2.19)	< 0.0001
Rheumatoid arthritis	254 (4.03)	155 (2.46)	< 0.0001
Sjogren's syndrome	110 (1.74)	71 (1.13)	0.003
Psoriasis	94 (1.49)	83 (1.32)	0.40
Ankylosing spondylitis	53 (0.84)	39 (0.62)	0.14
Hashimoto's thyroiditis	13 (0.21)	10 (0.16)	0.53
T1DM	78 (1.24)	68 (1.08)	0.40
T2DM	1473 (23.3)	1068 (16.9)	< 0.0001
Gout	1196 (18.9)	702 (11.1)	< 0.0001
Dyslipidemia	2252 (35.7)	1356 (21.5)	< 0.0001
Renal disease	585 (9.28)	427 (6.77)	< 0.0001

*CFS* chronic fatigue syndrome, *T1DM* type 1 diabetes mellitus, *T2DM* type 2 diabetes mellitus, *SD* standard deviation

^†^Student’s t-test

**Fig. 2:**
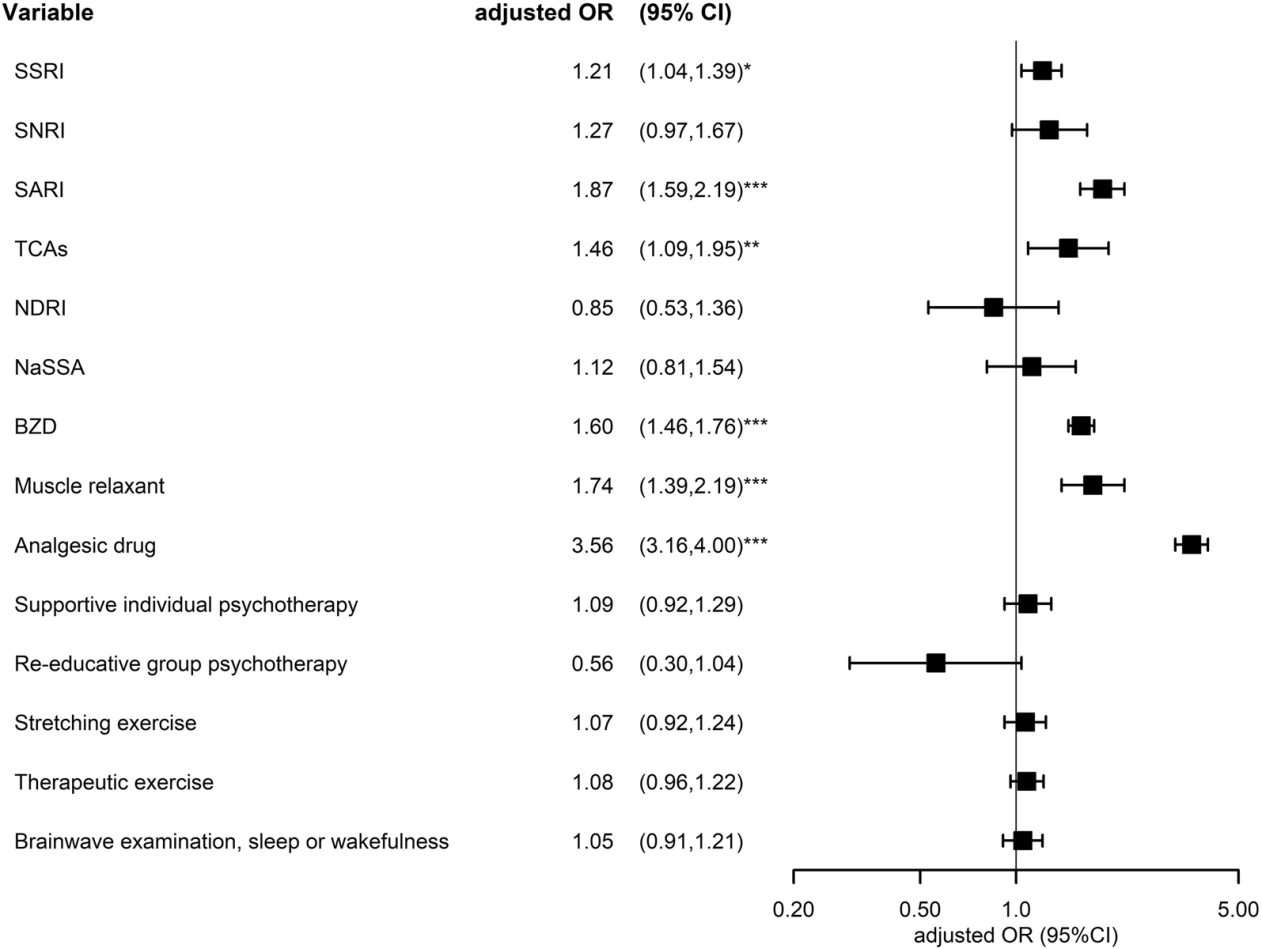
Forest plot of conditional logical regression measured odds ratios and 95% confidence interval of chronic fatigue syndrome with different treatments. *CFS* chronic fatigue syndrome, *CI* confidence interval, *SSRI* selective serotonin reuptake inhibitor, *SNRI* serotonin and norepinephrine reuptake inhibitor, *SARI* serotonin antagonist and reuptake inhibitor, *TCA* tricyclic antidepressants, *NDRI* norepinephrine and dopamine reuptake inhibitor, *NaSSA* noradrenergic and specific serotonergic antidepressants, *BZD* benzodiazepine; *P < .0.05, **P < .0.01, ***P < .001

Table 2 and Fig. 2 shows the pharmacological and no-pharmacological treatment received before the diagnosis of CFS. Participants taking certain types of antidepressants, including SSRI, SARI, and TCA, had higher odds of CSF, with the adjusted odds ratio (aORs) of 1.21 (95% CI 1.04–1.39), 1.87 (95% CI 1.59–2.19), and 1.46 (95% CI 1.09–1.95). Other drugs with increased aORs of CFS included BZDs (1.60, 95% CI 1.46–1.76), muscle relaxants (1.74, 95% CI 1.39–2.19), and analgesics (3.56, 95% CI 3.16–4). As for the non-pharmacological treatments and examinations received by the participants, undergoing brainwave examination had a significantly increased odds ratio (1.6, 95% CI 1.44–1.77) of CFS but an insignificant aOR after being adjusted with demographic data and comorbidities.

**Table 2:** Conditional logical regression measured odds ratios of chronic fatigue syndrome with different treatments

Variable	N	Control		CFS		Odds ratio				Multiple comparisons
		n	%	n	%	Crude (95% CI)	p-value	Adjusted (95% CI)	p-value	p-value
SSRI						1.90 (1.68,2.14)***	< 0.001	1.21 (1.04,1.39)*	0.011	0.001
No	11,365	5858	52%	5507	48%					
Yes	1247	448	36%	799	64%					
SNRI						2.11 (1.65,2.70)***	< 0.001	1.27 (0.97,1.67)	0.080	0.006
No	12,320	6211	50%	6109	50%					
Yes	292	95	33%	197	67%					
SARI						2.85 (2.46,3.31)***	< 0.001	1.87 (1.59,2.19)***	< 0.001	< 0.001
No	11,684	6052	52%	5632	48%					
Yes	928	254	27%	674	73%					
TCAs						2.04 (1.55,2.68)***	< 0.001	1.46 (1.09,1.95)**	0.010	0.001
No	12,374	6227	50%	6147	50%					
Yes	238	79	33%	159	67%					
NDRI						1.85 (1.20,2.85)**	0.005	0.85 (0.53,1.36)	0.495	0.035
No	12,521	6274	50%	6247	50%					
Yes	91	32	35%	59	65%					
NaSSA						2.04 (1.51,2.74)***	< 0.001	1.12 (0.81,1.54)	0.508	0.036
No	12,413	6240	50%	6173	50%					
Yes	199	66	33%	133	67%					
BZD						2.14 (1.98,2.32)***	< 0.001	1.60 (1.46,1.76)***	< 0.001	< 0.001
No	3680	2328	63%	1352	37%					
Yes	8932	3978	45%	4954	55%					
Muscle relaxant						2.16 (1.74,2.69)***	< 0.001	1.74 (1.39,2.19)***	< 0.001	< 0.001
No	12,229	6183	51%	6046	49%					
Yes	383	123	32%	260	68%					
Analgesic drug						4.43 (3.96,4.97)***	< 0.001	3.56 (3.16,4.00)***	< 0.001	< 0.001
No	1995	1560	78%	435	22%					
Yes	10,617	4746	45%	5871	55%					
Supportive individual psychotherapy						1.10 (0.94,1.29)	0.229	1.09 (0.92,1.29)	0.302	0.022
No	11,956	5993	50%	5963	50%					
Yes	656	313	48%	343	52%					
Re-educative group psychotherapy						0.60 (0.33,1.08)	0.086	0.56 (0.30,1.04)	0.065	0.005
No	12,564	6276	50%	6288	50%					
Yes	48	30	63%	18	38%					
Stretching exercise						1.08 (0.94,1.25)	0.280	1.07 (0.92,1.24)	0.367	0.026
No	11,788	5909	50%	5879	50%					
Yes	824	397	48%	427	52%					
Therapeutic exercise						1.09 (0.98,1.22)	0.126	1.08 (0.96,1.22)	0.182	0.013
No	11,273	5663	50%	5610	50%					
Yes	1339	643	48%	696	52%					
Brainwave examination, sleep or wakefulness						1.60 (1.44,1.77)***	< 0.001	1.05 (0.91,1.21)	0.527	0.038
No	11,704	5864	50%	5840	50%					
Yes	908	442	49%	466	51%					

*CFS* chronic fatigue syndrome, *CI* confidence interval, *SSRI* selective serotonin reuptake inhibitor, *SNRI* serotonin and norepinephrine reuptake inhibitor, *SARI* serotonin antagonist and reuptake inhibitor, TCA tricyclic antidepressants, *NDRI* norepinephrine and dopamine reuptake inhibitor, *NaSSA* noradrenergic and specific serotonergic antidepressants, *BZD* benzodiazepine

^*^P < .05, **P < .01, ***P < .001

**Fig. 3:**
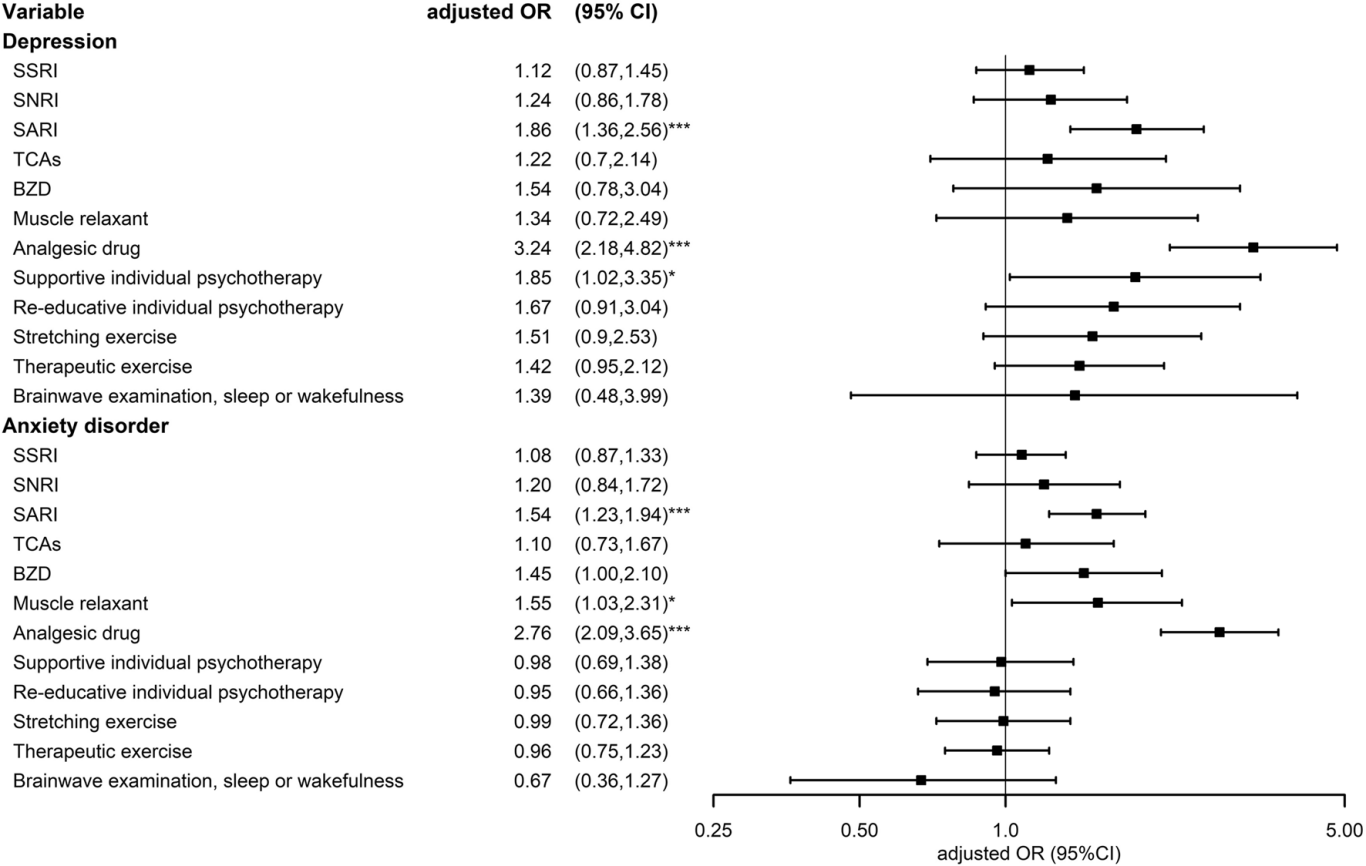
Forest plot of conditional logical regression measured odds ratios and 95% confidence interval of chronic fatigue syndrome with different treatments stratified by depression or anxiety disorder. *CFS* chronic fatigue syndrome, *CI* confidence interval, *SSRI* selective serotonin reuptake inhibitor, *SNRI* serotonin and norepinephrine reuptake inhibitor, *SARI* serotonin antagonist and reuptake inhibitor, *TCA* tricyclic antidepressants, *BZD* benzodiazepine; *P < 0.05, **P < .0.01., ***P < 0.001

Table 3 and Fig. 3 presents the treatment received before the diagnosis of chronic fatigue syndrome with comorbidity sub-classification by having depression or anxiety disorder. The aORs of SARI usages and analgesic drug usages increased in both groups with depression and anxiety disorders. Among the participants with depression who received supportive individual psychotherapy, the aORs of risk of CFS was 1.85 (95% CI 1.02–3.35). As for the participants with anxiety disorder, the aORs of risk of CFS was 1.55 (95% CI 1.03–2.31) in those who also take muscle relaxants.

**Table 3:** Conditional logical regression measured odds ratios of chronic fatigue syndrome with different treatments stratified by depression or anxiety disorder

Variable	Control		CFS		Odds ratio				Multiple comparisons
					Crude (95% CI)	p-value	Adjusted (95% CI)	p-value	p-value
	*Depression*								
	No	Yes	No	Yes					
SSRI					1.21 (0.95,1.54)	0.116	1.12 (0.87,1.45)	0.371	0.031
No	5656	202	5145	362					
Yes	243	205	354	445					
SNRI					1.38 (0.98,1.95)	0.065	1.24 (0.86,1.78)	0.253	0.021
No	5856	355	5438	671					
Yes	43	52	61	136					
SARI					2.04 (1.52,2.74)***	< 0.001	1.86 (1.36,2.56)***	< 0.001	< 0.001
No	5717	335	5071	561					
Yes	182	72	428	246					
TCAs					1.33 (0.78,2.26)	0.288	1.22 (0.70,2.14)	0.483	0.040
No	5840	387	5392	755					
Yes	59	20	107	52					
BZD					1.65 (0.88,3.11)	0.121	1.54 (0.78,3.04)	0.209	0.017
No	2310	18	1330	22					
Yes	3589	389	4169	785					
Muscle relaxant					1.73 (0.96,3.11)	0.07	1.34 (0.72,2.49)	0.351	0.029
No	5791	392	5289	757					
Yes	108	15	210	50					
Analgesic drug					3.61 (2.49,5.23)***	< 0.001	3.24 (2.18,4.82)***	< 0.001	< 0.001
No	1479	81	383	52					
Yes	4420	326	5116	755					
Supportive individual psychotherapy					1.68 (0.96,2.93)	0.069	1.85 (1.02,3.35)*	0.044	0.004
No	5603	390	5211	752					
Yes	296	17	288	55					
Re-educative individual psychotherapy					1.55 (0.88,2.72)	0.128	1.67 (0.91,3.04)	0.097	0.008
No	5632	390	5226	756					
Yes	267	17	273	51					
Stretching exercise					1.46 (0.89,2.39)	0.13	1.51 (0.90,2.53)	0.122	0.010
No	5525	384	5137	742					
Yes	374	23	362	65					
Therapeutic exercise					1.39 (0.94,2.04)	0.096	1.42 (0.95,2.12)	0.092	0.008
No	5296	367	4909	701					
Yes	603	40	590	106					
Brainwave examination, sleep or wakefulness					1.73 (0.63,4.72)	0.285	1.39	(0.48,3.99)	0.543
No	5781	402	5412	790					
Yes	118	5	87	17					

	Anxiety disorder								
	No	Yes	No	Yes					
SSRI					1.27 (1.06,1.51)**	0.009	1.08 (0.87,1.33)	0.486	0.041
No	5051	807	4003	1504					
Yes	222	226	265	534					
SNRI					1.40 (1.01,1.93)*	0.044	1.20 (0.84,1.72)	0.306	0.026
No	5231	980	4214	1895					
Yes	42	53	54	143					
SARI					1.80 (1.46,2.23)***	< 0.001	1.54 (1.23,1.94)***	< 0.001	< 0.001
No	5148	904	4011	1621					
Yes	125	129	257	417					
TCAs					1.26 (0.84,1.87)	0.264	1.10 (0.73,1.67)	0.639	0.053
No	5229	998	4195	1952					
Yes	44	35	73	86					
BZD					1.60 (1.12,2.28)**	0.009	1.45 (1.00,2.10)	0.051	0.004
No	2270	58	1279	73					
Yes	3003	975	2989	1965					
Muscle relaxant					1.71 (1.16,2.53)**	0.007	1.55 (1.03,2.31)*	0.034	0.003
No	5184	999	4120	1926					
Yes	89	34	148	112					
Analgesic drug					2.96 (2.26,3.87)***	< 0.001	2.76 (2.09,3.65)***	< 0.001	< 0.001
No	1422	138	334	101					
Yes	3851	895	3934	1937					
Supportive individual psychotherapy					0.95 (0.68,1.32)	0.749	0.98 (0.69,1.38)	0.892	0.074
No	5015	978	4028	1935					
Yes	258	55	240	103					
Re-educative individual psychotherapy					0.92 (0.65,1.30)	0.648	0.95 (0.66,1.36)	0.770	0.064
No	5041	981	4039	1943					
Yes	232	52	229	95					
Stretching exercise					0.97 (0.72,1.32)	0.867	0.99 (0.72,1.36)	0.961	0.080
No	4943	966	3970	1909					
Yes	330	67	298	129					
Therapeutic exercise					0.96 (0.76,1.23)	0.767	0.96 (0.75,1.23)	0.739	0.062
No	4740	923	3782	1828					
Yes	533	110	486	210					
Brainwave examination, sleep or wakefulness					0.64 (0.35,1.17)	0.144	0.67 (0.36,1.27)	0.221	0.018
No	5169	1014	4188	2014					
Yes	104	19	80	24					

*CFS* chronic fatigue syndrome, *CI* confidence interval, *SSRI* selective serotonin reuptake inhibitor, *SNRI* serotonin and norepinephrine reuptake inhibitor, *SARI* serotonin antagonist and reuptake inhibitor, *TCA* tricyclic antidepressants, *BZD* benzodiazepine

^*^P < .05, **P < .01, ***P < .001

**Fig. 4:**
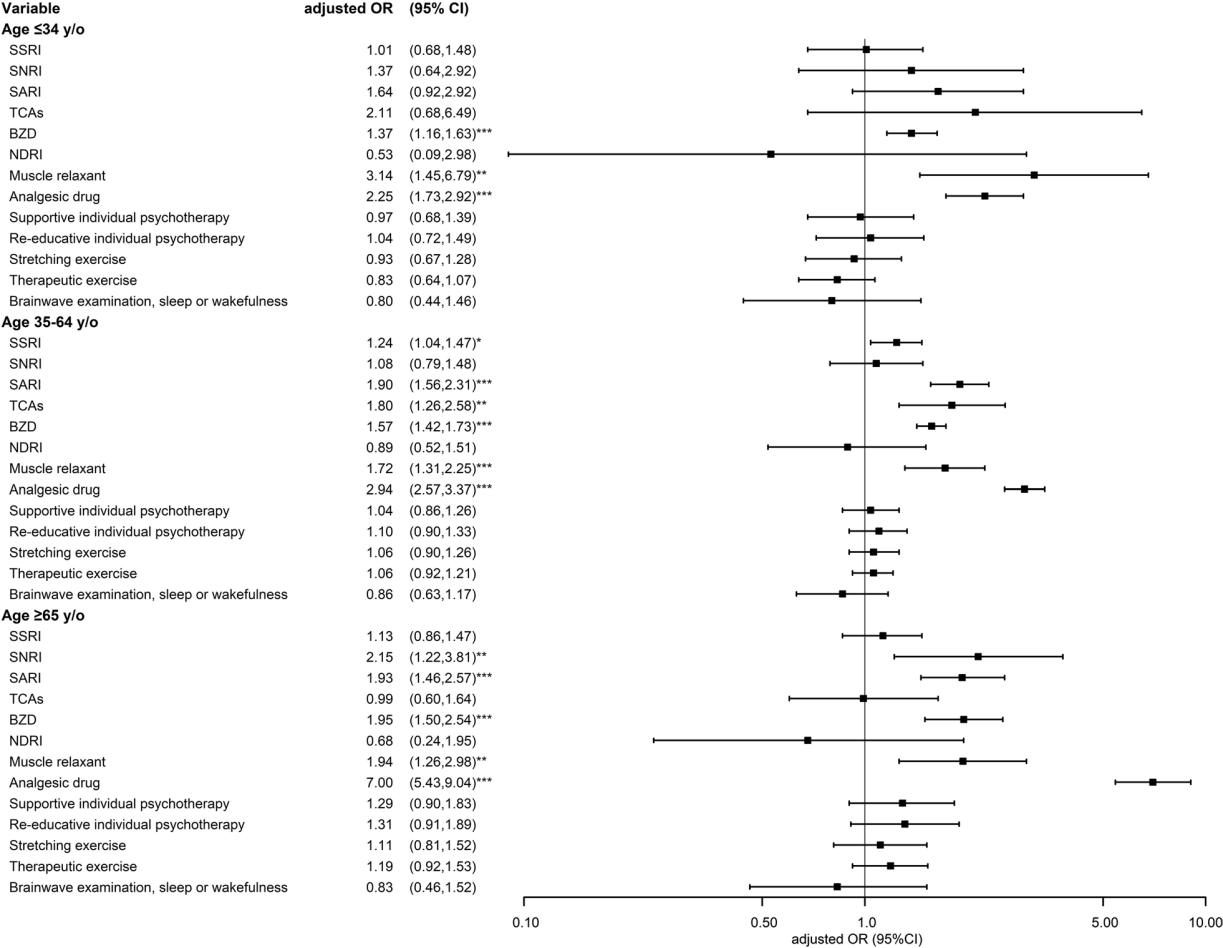
Forest plot of conditional logical regression measured odds ratios and 95% confidence interval of chronic fatigue syndrome with different treatments stratified by age. *CFS* chronic fatigue syndrome, *CI* confidence interval, *SSRI* selective serotonin reuptake inhibitor, *SNRI* serotonin and norepinephrine reuptake inhibitor, *SARI* serotonin antagonist and reuptake inhibitor, *TCA* tricyclic antidepressants, *BZD* benzodiazepine; *P < .0.05, **P < .0.01, ***P < .0.001

As presented in Table 4 and Fig. 4, the analysis with sub-classification by age also demonstrates different patterns of medications used across different ages. BZD, muscle relaxants, and analgesic drug usages were indicated on increased aORs of risks of CFS in all the age groups. In contrast, the usages of SSRI, SARI, and TCA among participants aging from 35 to 64 years old had aORs of 1.24 (95% CI 1.04–1.47), 1.90 (95% CI 1.56–2.31), and 1.80 (95% CI 1.26–2.58), respectively. Among participants aging over 65 years old, the use of serotonin and norepinephrine reuptake inhibitor (SNRI) and SARI, with aORs being 2.15 (95% CI 1.22–3.81) and 1.93 (95% CI 1.46–2.57), respectively.

**Table 4:** Conditional logical regression measured odds ratios of chronic fatigue syndrome with different treatments stratified by age

Variable	Control		CFS		Odds ratio				Multiple comparisons
					Crude (95% CI)	p-value	Adjusted (95% CI)	p-value	p-value
	*Age* ≤ *34 y/o*								
	No	Yes	No	Yes					
SSRI					1.72 (1.26,2.35)***	< 0.001	1.01 (0.68,1.48)	0.977	0.075
No	4678	1180	4367	1140					
Yes	380	68	686	113					
SNRI					2.61 (1.34,5.11)**	0.005	1.37 (0.64,2.92)	0.413	0.032
No	4975	1236	4887	1222					
Yes	83	12	166	31					
SARI					3.03 (1.79,5.12)***	< 0.001	1.64 (0.92,2.92)	0.095	0.007
No	4823	1229	4435	1197					
Yes	235	19	618	56					
TCAs					3.21 (1.17,8.80)*	0.023	2.11 (0.68,6.49)	0.194	0.015
No	4984	1243	4910	1237					
Yes	74	5	143	16					
BZD					1.69 (1.44,1.98)***	< 0.001	1.37 (1.16,1.63)***	< 0.001	< 0.001
No	1625	703	809	543					
Yes	3433	545	4244	710					
NDRI					2.67 (0.71,10.07)	0.148	0.53 (0.09,2.98)	0.473	0.036
No	5029	1245	5002	1245					
Yes	29	3	51	8					
Muscle relaxant					3.61 (1.71,7.59)***	< 0.001	3.14 (1.45,6.79)**	0.004	< 0.001
No	4944	1239	4825	1221					
Yes	114	9	228	32					
Analgesic drug					2.37 (1.84,3.05)***	< 0.001	2.25 (1.73,2.92)***	< 0.001	< 0.001
No	1349	211	336	99					
Yes	3709	1037	4717	1154					
Supportive individual psychotherapy					0.97 (0.69,1.36)	0.843	0.97 (0.68,1.39)	0.884	0.068
No	4816	1177	4779	1184					
Yes	242	71	274	69					
Re-educative individual psychotherapy					1.03 (0.72,1.46)	0.875	1.04 (0.72,1.49)	0.849	0.065
No	4838	1184	4795	1187					
Yes	220	64	258	66					
Stretching exercise					0.92 (0.68,1.26)	0.615	0.93 (0.67,1.28)	0.659	0.051
No	4749	1160	4708	1171					
Yes	309	88	345	82					
Therapeutic exercise					0.82 (0.64,1.06)	0.133	0.83 (0.64,1.07)	0.152	0.012
No	4564	1099	4483	1127					
Yes	494	149	570	126					
Brainwave examination, sleep or wakefulness					0.80 (0.45,1.43)	0.454	0.80 (0.44,1.46)	0.473	0.036
No	4961	1222	4970	1232					
Yes	97	26	83	21					

	*Age 35–64 y/o*								
	No	Yes	No	Yes					
SSRI					1.97 (1.71,2.27)***	< 0.001	1.24 (1.04,1.47)*	0.016	0.001
No	1338	4520	1256	4251					
Yes	133	315	215	584					
SNRI					1.84 (1.39,2.44)***	< 0.001	1.08 (0.79,1.48)	0.619	0.048
No	1453	4758	1414	4695					
Yes	18	77	57	140					
SARI					2.93 (2.45,3.51)***	< 0.001	1.90 (1.56,2.31)***	< 0.001	< 0.001
No	1386	4666	1261	4371					
Yes	85	169	210	464					
TCAs					2.41 (1.71,3.38)***	< 0.001	1.80 (1.26,2.58)**	0.001	< 0.001
No	1440	4787	1426	4721					
Yes	31	48	45	114					
BZD					2.12 (1.94,2.31)***	< 0.001	1.57 (1.42,1.73)***	< 0.001	< 0.001
No	268	2060	97	1255					
Yes	1203	2775	1374	3580					
NDRI					1.93 (1.19,3.13)**	0.008	0.89 (0.52,1.51)	0.655	0.050
No	1464	4810	1460	4787					
Yes	7	25	11	48					
Muscle relaxant					2.09 (1.61,2.70)***	< 0.001	1.72 (1.31,2.25)***	< 0.001	< 0.001
No	1437	4746	1393	4653					
Yes	34	89	78	182					
Analgesic drug					3.62 (3.18,4.12)***	< 0.001	2.94 (2.57,3.37)***	< 0.001	< 0.001
No	523	1037	96	339					
Yes	948	3798	1375	4496					
Supportive individual psychotherapy					1.04 (0.87,1.25)	0.648	1.04 (0.86,1.26)	0.657	0.051
No	1406	4587	1386	4577					
Yes	65	248	85	258					
Re-educative individual psychotherapy					1.09 (0.91,1.32)	0.343	1.10 (0.90,1.33)	0.350	0.027
No	1411	4611	1391	4591					
Yes	60	224	80	244					
Stretching exercise					1.06 (0.90,1.24)	0.512	1.06 (0.90,1.26)	0.475	0.037
No	1384	4525	1370	4509					
Yes	87	310	101	326					
Therapeutic exercise					1.06 (0.93,1.20)	0.411	1.06 (0.92,1.21)	0.408	0.031
No	1332	4331	1304	4306					
Yes	139	504	167	529					
Brainwave examination, sleep or wakefulness					0.83 (0.62,1.12)	0.227	0.86 (0.63,1.17)	0.337	0.026
No	1445	4738	1448	4754					
Yes	26	97	23	81					

	*Age* ≥ *65 y/o*								
	No	Yes	No	Yes					
SSRI					1.72 (1.37,2.17)***	< 0.001	1.13 (0.86,1.47)	0.379	0.029
No	4520	1338	4251	1256					
Yes	315	133	584	215					
SNRI					3.25 (1.91,5.55)***	< 0.001	2.15 (1.22,3.81)**	0.009	0.001
No	4758	1453	4695	1414					
Yes	77	18	140	57					
SARI					2.72 (2.09,3.53)***	< 0.001	1.93 (1.46,2.57)***	< 0.001	< 0.001
No	4666	1386	4371	1261					
Yes	169	85	464	210					
TCAs					1.47 (0.92,2.33)	0.106	0.99 (0.60,1.64)	0.981	< 0.001
No	4787	1440	4721	1426					
Yes	48	31	114	45					
BZD					3.16 (2.47,4.03)***	< 0.001	1.95 (1.5,2.54)***	< 0.001	< 0.001
No	2060	268	1255	97					
Yes	2775	1203	3580	1374					
NDRI					1.58 (0.61,4.08)	0.348	0.68 (0.24,1.95)	0.475	0.037
No	4810	1464	4787	1460					
Yes	25	7	48	11					
Muscle relaxant					2.37 (1.57,3.56)***	< 0.001	1.94 (1.26,2.98)**	0.003	< 0.001
No	4746	1437	4653	1393					
Yes	89	34	182	78					
Analgesic drug					7.90 (6.26,9.97)***	< 0.001	7.00 (5.43,9.04)***	< 0.001	< 0.001
No	1037	523	339	96					
Yes	3798	948	4496	1375					
Supportive individual psychotherapy					1.33 (0.95,1.85)	0.095	1.29 (0.90,1.83)	0.161	0.012
No	4587	1406	4577	1386					
Yes	248	65	258	85					
Re-educative individual psychotherapy					1.35 (0.96,1.91)	0.084	1.31 (0.91,1.89)	0.144	0.011
No	4611	1411	4591	1391					
Yes	224	60	244	80					
Stretching exercise					1.17 (0.87,1.58)	0.292	1.11 (0.81,1.52)	0.512	0.039
No	4525	1384	4509	1370					
Yes	310	87	326	101					
Therapeutic exercise					1.23 (0.97,1.56)	0.091	1.19 (0.92,1.53)	0.186	0.014
No	4331	1332	4306	1304					
Yes	504	139	529	167					
Brainwave examination, sleep or wakefulness					0.88 (0.50,1.55)	0.666	0.83 (0.46,1.52)	0.547	0.042
No	4738	1445	4754	1448					
Yes	97	26	81	23					

*CFS* chronic fatigue syndrome, *CI* confidence interval, *SSRI* selective serotonin reuptake inhibitor, *SNRI* serotonin and norepinephrine reuptake inhibitor, *SARI* serotonin antagonist and reuptake inhibitor, *TCA* tricyclic antidepressants, *BZD* benzodiazepine

^*^P < .05, **P < .01, ***P < .001

**Fig. 5:**
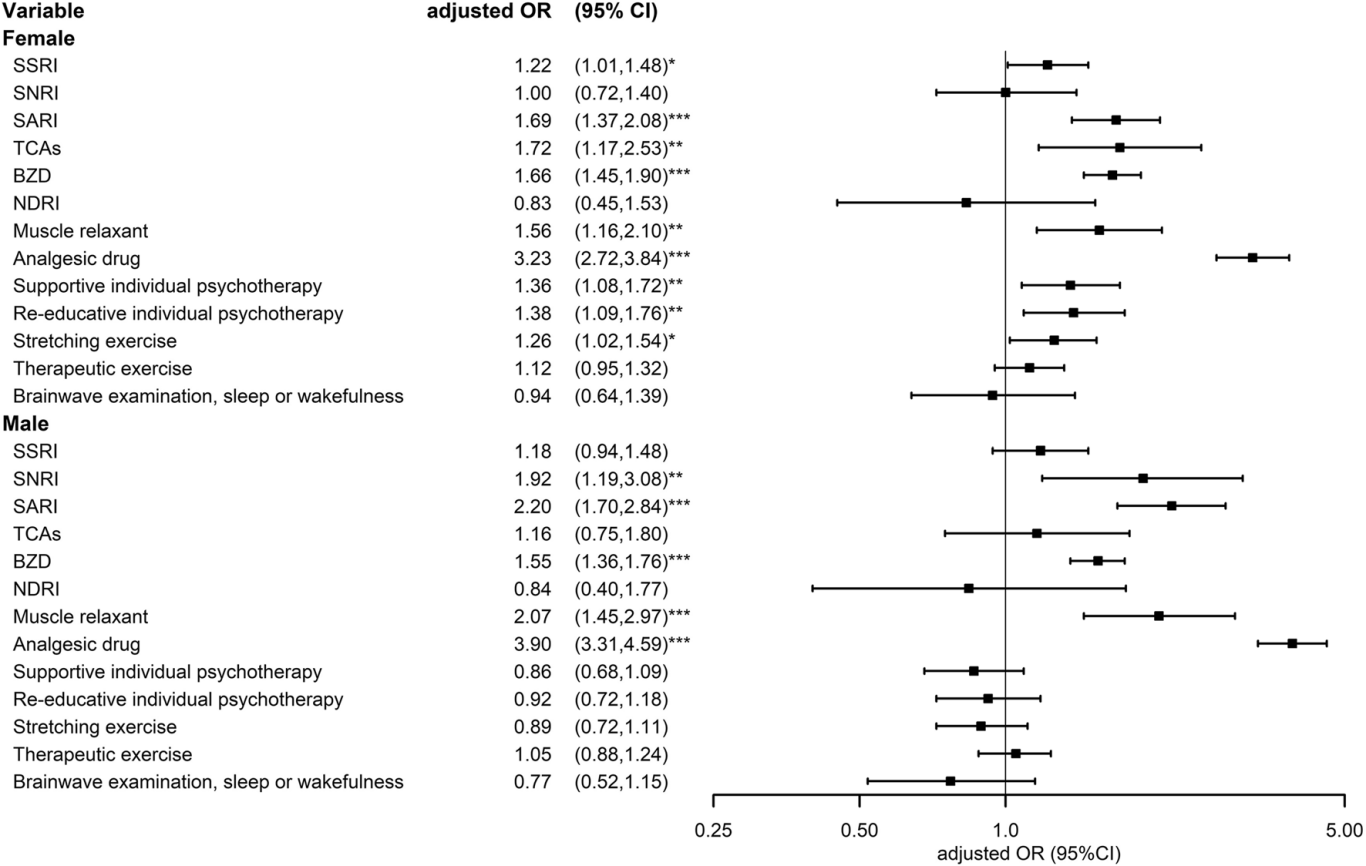
Forest plot of conditional logical regression measured odds ratios and 95% confidence interval of chronic fatigue syndrome with different treatments stratified by sex. *CFS* chronic fatigue syndrome, *CI* confidence interval, *SSRI* selective serotonin reuptake inhibitor, *SNRI* serotonin and norepinephrine reuptake inhibitor, *SARI* serotonin antagonist and reuptake inhibitor, *TCA* tricyclic antidepressants, *BZD* benzodiazepine; *P < .0.05, **P < .0.01, ***P < .0.001

In Table 5 and Fig. 5, we present the therapeutic options received by the patients with CFS and controls with sex specific sub-classification. In female patients, the adjusted odds ratio of risk of CFS were 1.22 (95% CI 1.01–1.48), 1.69 (95% CI 1.37–2.08), 1.72 (95% CI 1.17–2.53), 1.66 (95% CI 1.45–1.9), 1.56 (95% CI 1.16–2.1), 3.23 (95% CI 2.72–3.84), 1.36 (95% CI 1.08–1.72), 1.38 (95% CI 1.09–1.76), and 1.26 (95% CI 1.02–1.54), folds with SSRI use, SARI use, TCA use, BZD use, muscle relaxant use, analgesic drug use, supportive individual psychotherapy, re-educative psychotherapy, and stretching exercise. In male patients, the adjusted odds ratio risk of CFS were 1.92 (95% CI 1.19–3.08), 2.20 (95% CI 1.70–2.84), 1.55 (95% CI 1.36–1.76), 2.07 (95% CI 1.45–2.97), and 3.90 (95% CI 3.31–4.59) folds with SNRI use, SARI use, BZD use, muscle relaxant use, and analgesic drug use.

**Table 5:** Conditional logical regression measured odds ratios and 95% confidence interval of chronic fatigue syndrome with different treatments stratified by sex

Variable	Control		CFS		Odds ratio				multiple comparisons
	*Female*				Crude (95% CI)	p-value	Adjusted (95% CI)	p-value	p-value
	No	Yes	No	Yes					
SSRI					1.95 (1.67,2.29)***	< 0.001	1.22 (1.01,1.48)*	0.035	0.003
No	2785	3073	2651	2856					
Yes	182	266	316	483					
SNRI					1.70 (1.25,2.31)***	< 0.001	1.00 (0.72,1.40)	0.982	0.076
No	2940	3271	2884	3225					
Yes	27	68	83	114					
SARI					2.57 (2.12,3.12)***	< 0.001	1.69 (1.37,2.08)***	< 0.001	< 0.001
No	2871	3181	2671	2961					
Yes	96	158	296	378					
TCAs					2.46 (1.7,3.55)***	< 0.001	1.72 (1.17,2.53)**	0.006	0.000
No	2929	3298	2907	3240					
Yes	38	41	60	99					
BZD					2.28 (2.03,2.56)***	< 0.001	1.66 (1.45,1.9)***	< 0.001	< 0.001
No	1288	1040	799	553					
Yes	1679	2299	2168	2786					
NDRI					1.80 (1.02,3.16)*	0.041	0.83 (0.45,1.53)	0.553	0.043
No	2954	3320	2942	3305					
Yes	13	19	25	34					
Muscle relaxant					1.98 (1.49,2.62)***	< 0.001	1.56 (1.16,2.10)**	0.004	0.000
No	2919	3264	2852	3194					
Yes	48	75	115	145					
Analgesic drug					4.12 (3.49,4.86)***	< 0.001	3.23 (2.72,3.84)***	< 0.001	< 0.001
No	860	700	233	202					
Yes	2107	2639	2734	3137					
Supportive individual psychotherapy					1.36 (1.09,1.70)**	0.006	1.36 (1.08,1.72)**	0.009	0.001
No	2797	3196	2816	3147					
Yes	170	143	151	192					
Re-educative individual psychotherapy					1.38 (1.10,1.74)**	0.006	1.38 (1.09,1.76)**	0.008	0.001
No	2815	3207	2823	3159					
Yes	152	132	144	180					
Stretching exercise					1.26 (1.04,1.54)*	0.02	1.26 (1.02,1.54)*	0.029	0.002
No	2763	3146	2780	3099					
Yes	204	193	187	240					
Therapeutic exercise					1.13 (0.97,1.32)	0.126	1.12 (0.95,1.32)	0.195	0.015
No	2649	3014	2634	2976					
Yes	318	325	333	363					
Brainwave examination, sleep or wakefulness					0.90 (0.62,1.30)	0.568	0.94 (0.64,1.39)	0.768	0.059
No	2903	3280	2916	3286					
Yes	64	59	51	53					

	*Male*								
	No	Yes	No	Yes					
SSRI					1.82 (1.51,2.21)***	< 0.001	1.18 (0.94,1.48)	0.165	0.013
No	3073	2785	2856	2651					
Yes	266	182	483	316					
SNRI					3.13 (2.02,4.85)***	< 0.001	1.92 (1.19,3.08)**	0.007	0.001
No	3271	2940	3225	2884					
Yes	68	27	114	83					
SARI					3.31 (2.62,4.20)***	< 0.001	2.20 (1.70,2.84)***	< 0.001	< 0.001
No	3181	2871	2961	2671					
Yes	158	96	378	296					
TCAs					1.59 (1.06,2.40)*	0.026	1.16 (0.75,1.80)	0.502	0.039
No	3298	2929	3240	2907					
Yes	41	38	99	60					
BZD					2.08 (1.87,2.32)***	< 0.001	1.55 (1.36,1.76)***	< 0.001	< 0.001
No	1040	1288	553	799					
Yes	2299	1679	2786	2168					
NDRI					1.93 (0.99,3.78)	0.055	0.84 (0.40,1.77)	0.640	0.049
No	3320	2954	3305	2942					
Yes	19	13	34	25					
Muscle relaxant					2.45 (1.74,3.45)***	< 0.001	2.07 (1.45,2.97)***	< 0.001	< 0.001
No	3264	2919	3194	2852					
Yes	75	48	145	115					
Analgesic drug					4.79 (4.10,5.59)***	< 0.001	3.90 (3.31,4.59)***	< 0.001	< 0.001
No	700	860	202	233					
Yes	2639	2107	3137	2734					
Supportive individual psychotherapy					0.88 (0.70,1.11)	0.276	0.86 (0.68,1.09)	0.225	0.017
No	3196	2797	3147	2816					
Yes	143	170	192	151					
Re-educative individual psychotherapy					0.94 (0.75,1.19)	0.633	0.92 (0.72,1.18)	0.527	0.041
No	3207	2815	3159	2823					
Yes	132	152	180	144					
Stretching exercise					0.91 (0.74,1.12)	0.374	0.89 (0.72,1.11)	0.306	0.024
No	3146	2763	3099	2780					
Yes	193	204	240	187					
Therapeutic exercise					1.05 (0.89,1.24)	0.533	1.05 (0.88,1.24)	0.592	0.046
No	3014	2649	2976	2634					
Yes	325	318	363	333					
Brainwave examination, sleep or wakefulness					0.79 (0.55,1.15)	0.222	0.77 (0.52,1.15)	0.201	0.015
No	3280	2903	3286	2916					
Yes	59	64	53	51					

*CFS* chronic fatigue syndrome, *CI* confidence interval, *SSRI* selective serotonin reuptake inhibitor, *SNRI* serotonin and norepinephrine reuptake inhibitor, *SARI* serotonin antagonist and reuptake inhibitor, *TCA* tricyclic antidepressants, *BZD* benzodiazepine

^*^P < .05, **P < .01, ***P < .001

## Discussions

Our nationwide population-based study revealed that sampled patients with CFS experienced more comorbidities, such as depression and anxiety. These findings are consistent with those of previous studies. Furthermore, the treatments received by the participants before their diagnosis of CFS were also explored, and the results indicated that the use of specific types of antidepressants (e.g., SSRI, SARI, and TCA) was related to an increased risk of a subsequent diagnosis of CFS. In addition, a subgroup analysis also revealed that the treatment received differed by comorbidities, age, and sex.

Notably, no clear male or female predominance was observed in the present study. Other studies have reported that the prevalence of CFS among female individuals was approximately two-fold higher than that among male individuals [1, 4, 16]. However, several studies from East Asia, including Japan and China, have reported almost 1:1 sex ratios with respect to CFS prevalence [17, 18]. Different definitions of cases led to the variations in the prevalence and the incidence of CFS. We defined CFS using the CDC-1994 criteria in this study since it is the most common one that may resulted in recruit more cases [4, 19]. Cross-cultural differences in diagnostic practices for CFS and other conditions, especially neurasthenia, could explain the aforementioned differences in reported findings [20, 21], and this could ultimately lead to partly dissimilar populations being diagnosed. Another possible cause is the accessibility of the healthcare systems in Taiwan, as the National Health Insurance had covered over 99.9% of the civilians [22]. The increased accessibility could decrease the numbers of undetected cases. It therefore highlights the importance of the detection of male patients with CFS who might potentially be neglected.

The demographic data (Table 1) of the participants of the present study indicated higher comorbidity rates of depression, anxiety, inflammatory bowel diseases (IBD; Crohn’s disease and ulcerative colitis), autoimmune diseases, and metabolic disorders relative to the general population. Studies have reported an association between metabolic syndrome and CFS and identified altered fatty acid levels and lipid metabolism in individuals with CFS through further plasma metabolic profiling [23–25]. Other studies have suggested the presence of a shared pathophysiological process in CFS, autoimmune rheumatic diseases, and inflammatory bowel diseases because of the reported associations among the conditions and their similar symptomatology [25–28]. The role of the immune system in CFS could also be highlighted by our previous findings of the correlation between CFS and infectious diseases, indicating the involvement of post-infection dysregulated immune response [29, 30]. These findings highlight the complexity of CFS and its potential causes.

The greater prevalence of depression and anxiety disorder among individuals with CFS is an extensively studied topic. In both adult and adolescent populations, a high comorbidity of depression and anxiety has been reported in the literature [6, 31, 32]. Similarly, our analysis revealed an almost two times higher prevalence of depression and anxiety disorders in addition to insomnia among the participants diagnosed with CFS (Table 1). The causal relationship between CFS and concurrent psychiatric disorders remains unclear. Several neuroimaging studies have produced similar findings (including decreased cortical glutathione levels and altered resting-state functional connectivity in the anterior cingulate cortex) in both individuals with CFS and individuals with depression [33–36], suggesting a shared pathophysiology.

The increased use of multiple types of antidepressants, especially SARI (mainly trazodone), has been observed before the diagnosis of CFS even after adjustments for clinical covariates, such as depression, anxiety, and insomnia (Tables 2 and 3.). In the diagnostic criteria for CFS, the applicable duration for defining unexplainable fatigue is a period in excess of 6 months [3], thus the prescription received by a patient at the point of diagnosis may correspond to the ongoing symptoms of CFS itself. Therefore, the medications prescribed during the aforementioned period may also provide us with a general overview of a patient’s status at the beginning of the clinical course of CFS.

In a clinical setting, trazodone is not only used as an antidepressant but also an efficacious treatment for insomnia at a low dose. Trazodone has been demonstrated to improve perceived sleep quality and reduce the number of early awakenings [37]. In Taiwan, trazodone is the fifth most frequently prescribed psychotropic drug in the outpatient clinics and has usually been used as a hypnotic [38]. In addition, it is also used off-label for anxiety and fibromyalgia in limited clinical settings [39]. SARI is speculated to be prescribed more frequently in such populations because of the accompanying subclinical symptoms of CFS, which include depression, anxiety, insomnia, and muscle pain [25]. This viewpoint is further supported by our finding regarding the increased pre diagnostic use of BZD, muscle relaxants, and analgesic drugs across all age groups in the participants with CFS (Table 4). Among the aforementioned symptoms, depression, and pain have been reported to be associated with decreased quality of life and physical functioning [40, 41]. Our data revealed that these disabling symptoms may occur in the early stage of the clinical course of CFS, and physicians must thus be aware of them.

With regard to sex, the pattern of antidepressant use differed between male and female participants with CFS. Before receiving a diagnosis of CFS, female participants were more likely to be taking SSRI and TCAs, whereas male participants were more likely to be taking SNRIs. This could be related to the sex-specific symptomatology in CFS, such as the higher prevalence of insomnia in female individuals relative to male individuals [42], which could lead to the prescription of sedative medications (e.g., TCAs and specific SSRIs) [43]. Higher ORs for receiving psychotherapy and rehabilitation were also observed in female individuals relative to male individuals, which could indicate a higher rate of engagement with medical services among female individuals with CFS and an insufficient awareness of CFS among male individuals. Similar sex differences have also been observed for other conditions, such as posttraumatic stress disorder and depression [44–46].

It is noticeable that, in younger groups, an increased risk of CFS is mainly associated with the usage of muscle relaxants and analgesics, rather than anti-depressants (shown in Table 5 and Fig. 5). Muscle pain is a common symptom of CFS, and some researchers even describe that CFS is “old muscle in young body [47].” Furthermore, adolescents with CFS were indicated to have lower pain thresholds [48]. In the present study, CFS patients suffer from muscle pain symptoms more than control participants do, so the increased use of muscle relaxants and analgesics before diagnosis in CFS was noted. We further analyzed whether there was a gender difference in this group (age < 34y) and found that in younger females, the use of BZD and analgesics was related to subsequent CFS diagnosis (Additional file 2: Table S1 and Additional file 1: Figure S1). In males, in addition to BZD and analgesics, SNRI and muscle relaxants were also related to an increased risk of subsequent CFS. The phenomena suggest that compared to young females, young males have more diverse symptoms before CFS onset, leading to more varieties of medications being prescribed.

Our previous study analyzed both pharmacological and nonpharmacological treatments administered after the diagnosis of CFS. In contrast to the present study, we noted an increased use of antidepressants with dual-targeting mechanisms (serotonin– noradrenaline reuptake inhibitors and norepinephrine–dopamine reuptake inhibitors) after a diagnosis [49]. Such medications have relatively well-established effects on fatigue and pain under multiple conditions [50–54]. As for nonpharmacological treatments, the number of patients receiving supportive psychotherapy, re-educative group psychotherapy, stretching exercise, and therapeutic exercise significantly increased after, but not before, diagnosis of CFS [49]. The contrast between these two studies indicates the extensive and multimodal approach taken in the Taiwanese health care system in treating CFS.

Studies have increasingly demonstrated the long-term postinfection symptoms of COVID-19, a phenomenon termed long COVID. The symptoms include persistent fatigue, pain, postexertional malaise, and appetite loss [55, 56]. Because the symptomatology of long COVID indicates certain similarities to that of CFS, a shared pathophysiology may be possible, such as alterations in oxidative stress or the hypothalamic–pituitary–adrenal (HPA) axis [57–59]. Our results may also contribute to investigations into identifying populations that are at high risk of long COVID. One study showed that female sex is a risk factor for long COVID [55]. Another preliminary study focusing on patients with multiple sclerosis demonstrated that pre-existing depression and anxiety were associated with increased risk of long COVID [60]. These findings accord with our findings regarding CFS. The increased susceptibility to CFS and long COVID among these populations might be related to depression-related or anxiety-related increases in oxidative stress [61, 62] or HPA axis dysregulation [63, 64]. Because the research on this topic is limited, further studies should compare the mechanisms of CFS and long COVID and investigate the implications for prevention and treatment.

This study has several limitations. First, the associations between CFS and the severity of depression and anxiety were not classified. Furthermore, due to the nature of the datasets from the NHIRD, the characteristics and the severity of the symptomatology in the patients were not recorded. The detailed associations between the medications prescribed and the severity of clinical symptoms couldn’t be investigated. As a results, the study aimed to speculate the corresponding symptomatology of the patients according to the genre of medications they received. Further prospective clinical studies focusing on the causal relationship and subgroup analysis were therefore warranted. Second, the present study could only examine a limited sample because the CDC-1994 diagnosis criteria for CFS (ICD-9-CM 780.71) were adopted for this study. These criteria mainly center on neurologic and neurocognitive symptoms; however, it did not incorporate other common accompanying symptoms, such as orthostatic intolerance, anorexia, and motor disturbance [65, 66], which are included in other newly proposed diagnostic criteria [19]. Therefore, the differences and similarities in the patterns of psychiatric comorbidities in CFS under different diagnostic criteria should be examined in future studies. Third, ethnic or geographic differences could not be clarified because the population examined in the present study mostly comprised East Asian individuals.

## Conclusion

This study is the first nationwide population-based study to report a higher risk of CFS in patients with depression and anxiety disorder, especially those taking SSRIs, SARIs, and TCAs. In addition, BZD, muscle relaxants, and analgesic drugs were also revealed to be indicators of an elevated risk of CFS. These findings can increase the awareness of clinicians regarding high-risk populations and extend our current understanding of CFS.

## Supplementary Information

**Additional file 1: Figure S1.** Forest plot of conditional logical regression measured odds ratios and 95% confidence interval of chronic fatigue syndrome with different treatments stratified by sex in participants younger than 34 years old. CFS chronic fatigue syndrome, CI confidence interval, SSRI selective serotonin reuptake inhibitor, SNRI serotonin and norepinephrine reuptake inhibitor, SARI serotonin antagonist and reuptake inhibitor, TCA tricyclic antidepressants, BZD benzodiazepine; *P < .05, **P < .01, ***P < .001.

**Additional file 2: Table S1.** Conditional logical regression measured odds ratios and 95% confidence interval of chronic fatigue syndrome with different treatments stratified by sex in participants younger than 34 years old.

## Data Availability

The data underlying this study is from the National Health Insurance Research database (NHIRD). Interested researchers can obtain the data through formal application to the Ministry of Health and Welfare, Taiwan.
