# Supplementary figures and images for: Presence of depression and anxiety with distinct patterns of pharmacological treatments before the diagnosis of chronic fatigue syndrome: a population-based study in Taiwan
Source: J Transl Med. 2023 Feb 8;21:98. doi: 10.1186/s12967-023-03886-1 (PMC9907887; doi:10.1186/s12967-023-03886-1)

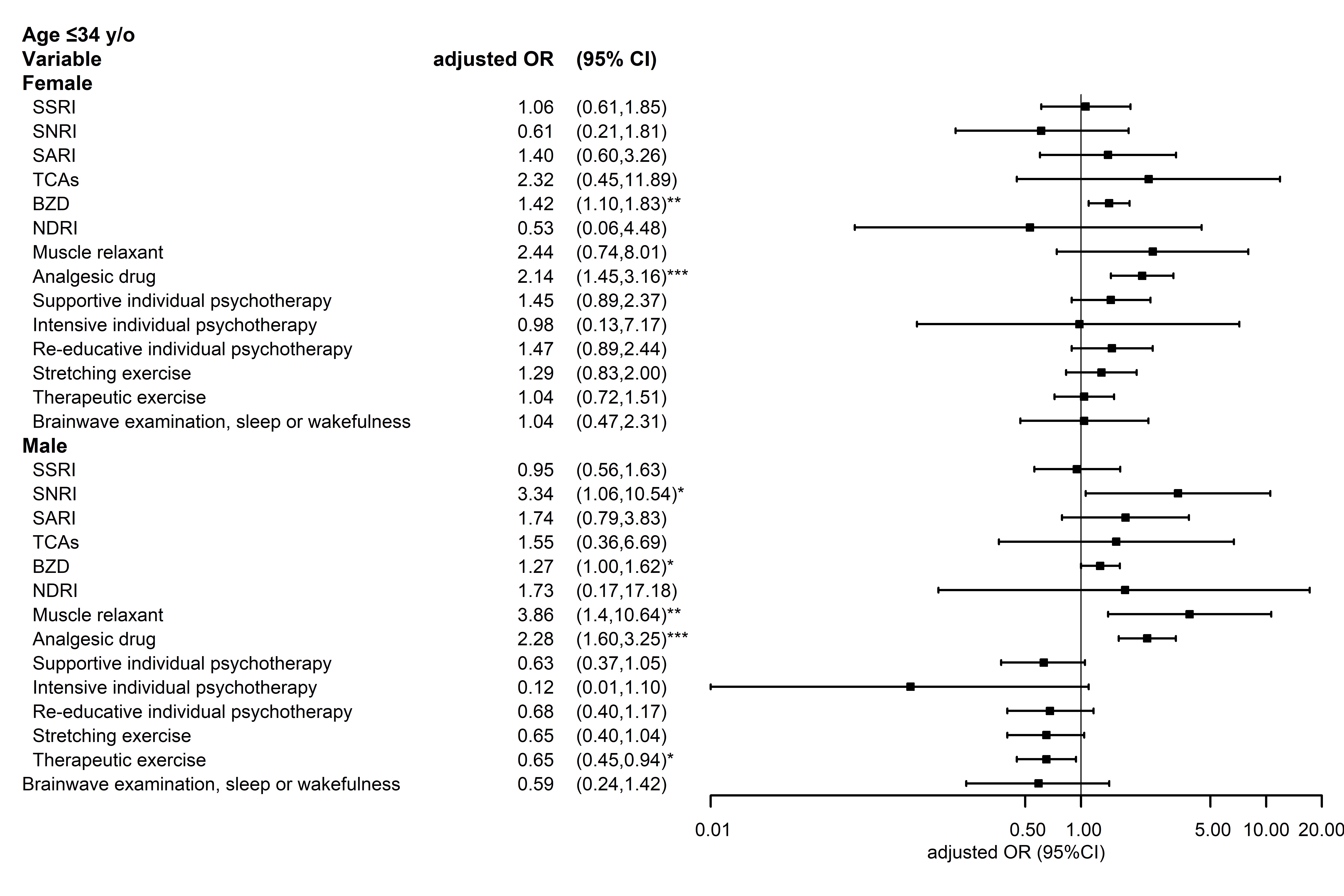

Supplement: Supplementary file 1 — Additional file 1: Figure S1. Forest plot of conditional logical regression measured odds ratios and 95% confidence interval of chronic fatigue syndrome with different treatments stratified by sex in participants younger than 34 years old. CFS chronic fatigue syndrome, CI confidence interval, SSRI selective serotonin reuptake inhibitor, SNRI serotonin and norepinephrine reuptake inhibitor, SARI serotonin antagonist and reuptake inhibitor, TCA tricyclic antidepressants, BZD benzodiazepine; *P < .05, **P < .01, ***P < .001. [file 12967_2023_3886_MOESM1_ESM.jpg]
