# Supplementary material for: Presence of depression and anxiety with distinct patterns of pharmacological treatments before the diagnosis of chronic fatigue syndrome: a population-based study in Taiwan
Source: J Transl Med. 2023 Feb 8;21:98. doi: 10.1186/s12967-023-03886-1 (PMC9907887; doi:10.1186/s12967-023-03886-1)
Supplement: Supplementary file 2 — Additional file 2: Table S1. Conditional logical regression measured odds ratios and 95% confidence interval of chronic fatigue syndrome with different treatments stratified by sex in participants younger than 34 years old. [file 12967_2023_3886_MOESM2_ESM.docx]

Table S1 Conditional logical regression measured odds ratios and 95% confidence interval of chronic fatigue syndrome with different treatments stratified by sex in participants younger than 34 years old

|  | Age ≤34 y/o | | | | |  |  |  |  |  |  |  |  |  |
| --- | --- | --- | --- | --- | --- | --- | --- | --- | --- | --- | --- | --- | --- | --- |
|  | Control | |  | CFS | |  | Odds ratio | | | | | | | multiple comparisons |
| Variable |  |  |  |  |  |  | crude | (95% CI) | p-value |  | adjusted | (95% CI) | p-value | p-value |
|  | *Female* | | | | |  |  |  |  |  |  |  |  |  |
|  | No | Yes |  | No | Yes |  |  |  |  |  |  |  |  |  |
| SSRI |  |  |  |  |  |  | 0.58 | (0.37,0.91)* | 0.017 |  | 1.06 | (0.61,1.85) | 0.838 | 0.060 |
| No | 615 | 565 |  | 594 | 546 |  |  |  |  |  |  |  |  |  |
| Yes | 35 | 33 |  | 58 | 55 |  |  |  |  |  |  |  |  |  |
| SNRI |  |  |  |  |  |  | 0.89 | (0.34,2.33) | 0.815 |  | 0.61 | (0.21,1.81) | 0.374 | 0.027 |
| No | 645 | 590 |  | 630 | 592 |  |  |  |  |  |  |  |  |  |
| Yes | 4 | 8 |  | 22 | 9 |  |  |  |  |  |  |  |  |  |
| SARI |  |  |  |  |  |  | 0.35 | (0.16,0.76)** | 0.008 |  | 1.40 | (0.6,3.26) | 0.440 | 0.031 |
| No | 640 | 589 |  | 621 | 579 |  |  |  |  |  |  |  |  |  |
| Yes | 10 | 9 |  | 31 | 25 |  |  |  |  |  |  |  |  |  |
| TCAs |  |  |  |  |  |  | 0.25 | (0.05,1.18) | 0.079 |  | 2.32 | (0.45,11.89) | 0.312 | 0.022 |
| No | 647 | 596 |  | 644 | 593 |  |  |  |  |  |  |  |  |  |
| Yes | 3 | 2 |  | 8 | 8 |  |  |  |  |  |  |  |  |  |
| BZD |  |  |  |  |  |  | 0.59 | (0.47,0.74)*** | <0.001 |  | 1.42 | (1.1,1.83)** | 0.007 | 0.001 |
| No | 397 | 306 |  | 395 | 302 |  |  |  |  |  |  |  |  |  |
| Yes | 253 | 292 |  | 257 | 299 |  |  |  |  |  |  |  |  |  |
| NDRI |  |  |  |  |  |  | 1.01 | (0.14,7.16) | 0.996 |  | 0.53 | (0.06,4.48) | 0.556 | 0.040 |
| No | 649 | 596 |  | 646 | 599 |  |  |  |  |  |  |  |  |  |
| Yes | 1 | 2 |  | 6 | 2 |  |  |  |  |  |  |  |  |  |
| Muscle relaxant |  |  |  |  |  |  | 0.33 | (0.11,1.03) | 0.056 |  | 2.44 | (0.74,8.01) | 0.142 | 0.010 |
| No | 645 | 594 |  | 632 | 589 |  |  |  |  |  |  |  |  |  |
| Yes | 5 | 4 |  | 20 | 12 |  |  |  |  |  |  |  |  |  |
| Analgesic drug |  |  |  |  |  |  | 0.43 | (0.3,0.63)*** | <0.001 |  | 2.14 | (1.45,3.16)*** | <0.001 | <0.001 |
| No | 117 | 94 |  | 54 | 45 |  |  |  |  |  |  |  |  |  |
| Yes | 533 | 504 |  | 598 | 556 |  |  |  |  |  |  |  |  |  |
| Supportive individual psychotherapy | | | | |  |  | 0.73 | (0.45,1.17) | 0.193 |  | 1.45 | (0.89,2.37) | 0.134 | 0.010 |
| No | 610 | 567 |  | 625 | 599 |  |  |  |  |  |  |  |  |  |
| Yes | 40 | 31 |  | 27 | 42 |  |  |  |  |  |  |  |  |  |
| Intensive individual psychotherapy | | | | |  |  | 1.01 | (0.14,7.16) | 0.996 |  | 0.98 | (0.13,7.17) | 0.985 | 0.070 |
| No | 645 | 596 |  | 651 | 599 |  |  |  |  |  |  |  |  |  |
| Yes | 5 | 2 |  | 1 | 2 |  |  |  |  |  |  |  |  |  |
| Re-educative individual psychotherapy | | | | | |  | 0.71 | (0.44,1.17) | 0.181 |  | 1.47 | (0.89,2.44) | 0.132 | 0.009 |
| No | 615 | 569 |  | 626 | 561 |  |  |  |  |  |  |  |  |  |
| Yes | 35 | 29 |  | 26 | 40 |  |  |  |  |  |  |  |  |  |
| Stretching exercise | |  |  |  |  |  | 0.83 | (0.54,1.28) | 0.396 |  | 1.29 | (0.83,2) | 0.266 | 0.019 |
| No | 603 | 557 |  | 619 | 552 |  |  |  |  |  |  |  |  |  |
| Yes | 47 | 41 |  | 33 | 49 |  |  |  |  |  |  |  |  |  |
| Therapeutic exercise | |  |  |  |  |  | 0.97 | (0.68,1.39) | 0.879 |  | 1.04 | (0.72,1.51) | 0.830 | 0.059 |
| No | 567 | 532 |  | 594 | 533 |  |  |  |  |  |  |  |  |  |
| Yes | 83 | 66 |  | 58 | 68 |  |  |  |  |  |  |  |  |  |
| Brainwave examination, sleep or wakefulness | | | | | | | 1.09 | (0.49,2.41) | 0.83 |  | 1.04 | (0.47,2.31) | 0.928 | 0.066 |
| No | 637 | 585 |  | 643 | 589 |  |  |  |  |  |  |  |  |  |
| Yes | 13 | 13 |  | 9 | 12 |  |  |  |  |  |  |  |  |  |
|  | *Male* | | | | |  |  |  |  |  |  |  |  |  |
|  | No | Yes |  | No | Yes |  |  |  |  |  |  |  |  |  |
| SSRI |  |  |  |  |  |  | 0.58 | (0.38,0.9)* | 0.015 |  | 0.95 | (0.56,1.63) | 0.862 | 0.062 |
| No | 565 | 615 |  | 546 | 594 |  |  |  |  |  |  |  |  |  |
| Yes | 33 | 35 |  | 55 | 58 |  |  |  |  |  |  |  |  |  |
| SNRI |  |  |  |  |  |  | 0.18 | (0.06,0.52)** | 0.002 |  | 3.34 | (1.06,10.54)* | 0.040 | 0.003 |
| No | 590 | 645 |  | 592 | 630 |  |  |  |  |  |  |  |  |  |
| Yes | 8 | 4 |  | 9 | 22 |  |  |  |  |  |  |  |  |  |
| SARI |  |  |  |  |  |  | 0.31 | (0.15,0.64)** | 0.002 |  | 1.74 | (0.79,3.83) | 0.166 | 0.012 |
| No | 589 | 640 |  | 579 | 621 |  |  |  |  |  |  |  |  |  |
| Yes | 9 | 10 |  | 25 | 31 |  |  |  |  |  |  |  |  |  |
| TCAs |  |  |  |  |  |  | 0.37 | (0.1,1.41) | 0.147 |  | 1.55 | (0.36,6.69) | 0.559 | 0.040 |
| No | 596 | 647 |  | 593 | 644 |  |  |  |  |  |  |  |  |  |
| Yes | 2 | 3 |  | 8 | 8 |  |  |  |  |  |  |  |  |  |
| BZD |  |  |  |  |  |  | 0.59 | (0.47,0.73)*** | <0.001 |  | 1.27 | (1,1.62)* | 0.048 | 0.003 |
| No | 306 | 397 |  | 302 | 395 |  |  |  |  |  |  |  |  |  |
| Yes | 292 | 253 |  | 299 | 257 |  |  |  |  |  |  |  |  |  |
| NDRI |  |  |  |  |  |  | 0.17 | (0.02,1.38) | 0.097 |  | 1.73 | (0.17,17.18) | 0.641 | 0.046 |
| No | 596 | 649 |  | 599 | 646 |  |  |  |  |  |  |  |  |  |
| Yes | 2 | 1 |  | 2 | 6 |  |  |  |  |  |  |  |  |  |
| Muscle relaxant |  |  |  |  |  |  | 0.24 | (0.09,0.66)** | 0.005 |  | 3.86 | (1.4,10.64)** | 0.009 | 0.001 |
| No | 594 | 645 |  | 589 | 632 |  |  |  |  |  |  |  |  |  |
| Yes | 4 | 5 |  | 12 | 20 |  |  |  |  |  |  |  |  |  |
| Analgesic drug |  |  |  |  |  |  | 0.41 | (0.29,0.58)*** | <0.001 |  | 2.28 | (1.6,3.25)*** | <0.001 | <0.001 |
| No | 94 | 117 |  | 45 | 54 |  |  |  |  |  |  |  |  |  |
| Yes | 504 | 533 |  | 556 | 598 |  |  |  |  |  |  |  |  |  |
| Supportive individual psychotherapy | | | | |  |  | 1.52 | (0.92,2.5) | 0.102 |  | 0.63 | (0.37,1.05) | 0.076 | 0.005 |
| No | 567 | 610 |  | 599 | 625 |  |  |  |  |  |  |  |  |  |
| Yes | 31 | 40 |  | 42 | 27 |  |  |  |  |  |  |  |  |  |
| Intensive individual psychotherapy | | | | |  |  | 5.05 | (0.59,43.3) | 0.14 |  | 0.12 | (0.01,1.1) | 0.060 | 0.004 |
| No | 596 | 645 |  | 599 | 651 |  |  |  |  |  |  |  |  |  |
| Yes | 2 | 5 |  | 2 | 1 |  |  |  |  |  |  |  |  |  |
| Re-educative individual psychotherapy | | | | | |  | 1.37 | (0.82,2.3) | 0.235 |  | 0.68 | (0.4,1.17) | 0.163 | 0.012 |
| No | 569 | 615 |  | 561 | 626 |  |  |  |  |  |  |  |  |  |
| Yes | 29 | 35 |  | 40 | 26 |  |  |  |  |  |  |  |  |  |
| Stretching exercise | |  |  |  |  |  | 1.46 | (0.92,2.31) | 0.105 |  | 0.65 | (0.4,1.04) | 0.073 | 0.005 |
| No | 557 | 603 |  | 552 | 619 |  |  |  |  |  |  |  |  |  |
| Yes | 41 | 47 |  | 49 | 33 |  |  |  |  |  |  |  |  |  |
| Therapeutic exercise | |  |  |  |  |  | 1.5 | (1.05,2.14)* | 0.025 |  | 0.65 | (0.45,0.94)* | 0.022 | 0.002 |
| No | 532 | 567 |  | 533 | 594 |  |  |  |  |  |  |  |  |  |
| Yes | 66 | 83 |  | 68 | 58 |  |  |  |  |  |  |  |  |  |
| Brainwave examination, sleep or wakefulness | | | | | | | 1.46 | (0.62,3.44) | 0.388 |  | 0.59 | (0.24,1.42) | 0.236 | 0.017 |
| No | 585 | 637 |  | 589 | 643 |  |  |  |  |  |  |  |  |  |
| Yes | 13 | 13 |  | 12 | 9 |  |  |  |  |  |  |  |  |  |
